# Screening of in vitro-produced cattle embryos to assess incidence and characteristics of unbalanced chromosomal aberrations

**DOI:** 10.3168/jdsc.2022-0275

**Published:** 2023-01-31

**Authors:** Aniek C. Bouwman, Erik Mullaart

**Affiliations:** 1 Animal Breeding & Genomics, Wageningen University & Research, PO Box 338, 6700 AH Wageningen, the Netherlands; 2 CRV B.V., Wassenaarweg 20, 6843 NW, Arnhem, the Netherlands

## Abstract

In cattle, pregnancy rates of in vitro-produced embryos are lower than those of in vivo-produced embryos. One of the reasons may be the increase in chromosomal aberrations due to in vitro maturation and fertilization of the oocyte. Currently, embryo transfer is commonly applied in nucleus cattle breeding programs, and the embryos are genotyped for genomic selection. Therefore, intensity data from SNP arrays can be exploited for preimplantation genetic testing by screening the intensity data of the embryos for unbalanced chromosomal aberrations. A total of 558 stage 8 Dutch Holstein embryos genotyped with SNP arrays were screened in an observational study in retrospect. We found a 5% incidence rate of unbalanced chromosomal aberrations (aneuploidy and ploidy issues) among 430 successfully genotyped cattle embryos. The 22 affected embryos showed either aneuploidy or ploidy issues; monosomy was most frequently observed (14/22). In most cases (16/19) the maternal chromosome or chromosomes were lost or gained. One of the monosomy cases gave rise to a live-born fully diploid individual, suggesting mosaicism. Given that embryo genotypes are readily available, monitoring incidence can easily be applied. Moreover, selection for euploid embryos may improve pregnancy rates for in vitro embryo transfer.

## Highlights

• Genotype intensity data of embryos usable for aneuploidy screening.
• If both parents are genotyped, the parental origin of the aneuploidy can be assigned.
• The incidence of aneuploidy in this dataset of stage 8 embryos was 5%.
• In most cases (16/19) the maternal chromosome(s) were lost or gained.

## Graphical Abstract

**Figure:**
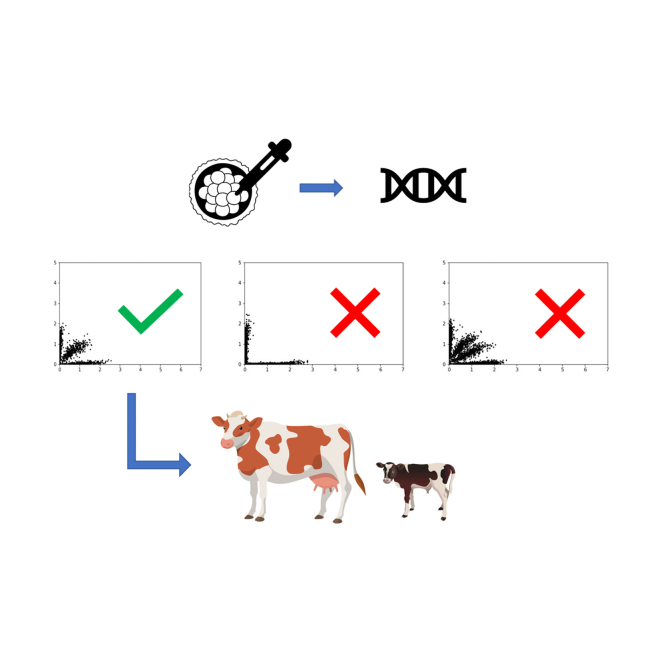
**Summary:** Pregnancy rates of in vitro-produced cattle embryos are lower than those of in vivo-produced embryos. One of the reasons may be the increase in chromosomal aberrations due to in vitro maturation and fertilization of the oocyte. Currently, embryo transfer is commonly applied in nucleus cattle breeding programs, and the embryos are genotyped for genomic selection. Therefore, intensity data from SNP arrays can be exploited for preimplantation genetic testing by screening the intensity data of the embryos for unbalanced chromosomal aberrations. A total of 558 stage 8 Dutch Holstein embryos genotyped with SNP arrays were screened in an observational study in retrospect. We found 22 affected embryos, showing either aneuploidy or ploidy issues; monosomy was most frequently observed. Given that embryo genotypes are readily available, preimplantation genetic screening can easily be applied to gain more insight in the characteristics of unbalanced chromosomal aberrations and their pregnancy outcomes.

In dairy breeding, embryo transfer has become an important technique to optimally exploit elite dams. Embryos can be flushed after superovulation and artificial insemination of a dam, referred to as in vivo embryo production. Or oocytes can be retrieved from a super ovulating dam by ovum pick-up, followed by artificial maturation and fertilization in the laboratory, referred to as in vitro embryo production. Both techniques are widely applied in practice, and a difference in pregnancy rates between embryos produced in vivo (64%) and in vitro (40%) has been observed (Ealy et al., 2019). One of the reasons could be the higher incidence of chromosomal aberrations observed in embryos produced in vitro (Tšuiko et al., 2017; Tutt et al., 2021). The chromosomal aberrations observed included whole-chromosome aberration (e.g., monosomy and trisomy), segmental aberrations (e.g., partial monosomy or trisomy), and aberrant ploidy level (e.g., haploid or triploid).

Chromosomal aberrations are the loss or gain of one or more chromosomes, or of a large segment thereof, and most forms are unviable. Currently, in nucleus breeding programs, embryos are genotyped with SNP arrays for selection on genomic breeding values (Mullaart and Wells, 2018). The generated genotype data can also be exploited to screen the embryos for such unbalanced aberrations before transfer. Large unbalanced aberrations can be detected using the intensity signals of the SNPs.

Silvestri et al. (2021) screened 1,713 embryo genotypes in a retrospect study, using a combination of log R ratio (**LRR**) and B allele frequency (**BAF**; Attiyeh et al., 2009), karyomapping (Handyside et al., 2010; Turner et al., 2019), and Gabriel–Griffin plots (Gabriel et al., 2011). They observed an incidence of chromosomal aberrations in 14% of the screened embryos, with the largest incidence in stage 5 embryos (24%), whereas the 103 stage 8 to 9 embryos showed only 2% incidence. Currently, stage 8 embryos are collected in routine practices, as these give the best results in terms of pregnancy success; hence, the overall incidence in practice may be as low as 2%. Therefore, we screened 558 stage 8 Holstein embryos genotyped with SNP arrays in retrospect to confirm the incidence rate of stage 8 embryos with a larger data set.

For this study, we focused on embryos produced in vitro from the cattle breeding program of the cattle improvement cooperative CRV (Arnhem, the Netherlands). Their routine system for embryo production entails hormonal stimulation of dams to induce superovulation to collect multiple oocytes per dam. The oocytes were collected with ovum pick-up, matured, and fertilized under laboratory conditions. Only grade 1 quality embryos, as categorized by the International Embryo Technology Society (Robertson, 1998), were used for biopsy. At blastocyst stage (stage 8), embryos were biopsied to retrieve around 10 cells from the trophoblast. The DNA was extracted from those cells and pre-amplified using a Single-Cell Repli-g Kit (Qiagen) to obtain sufficient DNA (at least a 10,000- to 20,000-fold amplification of the DNA; Mullaart and Wells, 2018) for genotyping.

Genotype data from 558 in vitro-produced embryos from 291 oocyte harvesting events from 2019 to 2020 were available. They were genotyped with either 20K SNPs or 50K SNPs from semi-custom Illumina arrays (Illumina Inc.). Final reports were extracted from Genome Studio 2.0 (Illumina Inc.) with all intensity data information: theta, R, LRR, BAF, and normalized X and Y intensity signal, as well as SNP quality information (GC score, GT score) and (AB) genotype call. We also extracted sample information from the DNA report, such as the call rate.

CRV keeps records of pregnancy outcomes following embryo transfer; however, only a limited number (181 out of 558) of the embryos were actually implanted in recipients. For those 181 transferred embryos, the pregnancy success and viability of the calf at birth (in case of successful pregnancy) were recorded. This information was only revealed to the researchers after the screening for unbalanced chromosomal aberrations was performed, to avoid bias in the screening. The data showed that 121 transferred embryos did not lead to a pregnancy. Of the 60 transferred embryos that did lead to a pregnancy, 55 calves were born alive, and 5 were born dead.

For every embryo, 3 different kinds of plots were created per chromosome. (1) The X and Y intensity levels of SNPs on the same chromosome were plotted against each other. In absence of chromosomal aberrations, the plot should show 3 clusters: one along the x-axis for AA genotypes, one along the y-axis for BB genotypes, and one along the diagonal for AB genotypes. If only 2 clusters were observed (along both axis; genotype A and B), the chromosome was classified as monosomic, and, if 4 clusters were observed, the chromosome was classified as trisomic (clusters for AAA, AAB, ABB, and BBB). (2) The BAF was plotted against the base pair position of the SNP on the chromosome. Here also, in absence of chromosomal aberrations, the plot should show 3 clusters: one around BAF of 0 for AA genotypes, one around BAF of 1 for BB genotypes, and one around BAF 0.5 for AB genotypes. If only 2 clusters were observed [BAF around 0 (A genotype) and 1 (B genotype)], the chromosome was classified as monosomic, and if 4 clusters were observed [BAF around 0 (AAA genotype), 0.33 (AAB genotype), 0.66 (ABB genotype), and 1 (BBB genotype)], the chromosome was classified as trisomic. (3) The LRR was plotted against the base pair position of the SNP on the chromosome. For monosomy cases, the LRR of the chromosome involved should be lower than on the other chromosomes. For trisomy cases, the LRR should be higher than the other chromosomes. For nullisomy (i.e., both copies of the particular chromosome are absent), LRR should be around −5, and BAF should be scattered all over the plot instead of forming the usual genotype clusters (Illumina, 2017). The BAF plots together with LLR plots could also indicate whether there is segmental aneuploidy affecting only part of the chromosome, because the positions of the SNPs are taken into account.

In a few cases (almost) all chromosomes were classified as either monosomic or trisomic, based on X and Y intensity and BAF plots, indicating a ploidy issue. When the ploidy level of an individual is affected, the LRR cannot be used as an indicator of copy number variant (Illumina, 2010). This is because the initial intensity values obtained are normalized within the sample itself; it is in later steps that clustering and calculation of R_expected_ (needed to calculate LRR) is done based on an external reference data set (Wang and Bucan, 2008). Due to this within-sample normalization, the LRR is not informative for copy number variation when the majority of the genome is not diploid (Illumina, 2010). Hence, the embryos were screened for ploidy issues based on only X and Y intensity and BAF plots.

The Y chromosome was not considered in this study because only a few SNP are located on the Y chromosome. The X chromosome was screened in terms of number of copies present, with none or more than 2 being abnormal. One X copy present was considered as normal, because male (XY) embryos have one copy of the X chromosome; hence, X− individuals remain undetected.

Furthermore, cases were confirmed using genotype data from the parents if available. For haploid and monosomy cases, SNPs with opposing homozygotes in the parents were investigated to see whether the embryo genotype matched only one of the parents across the whole chromosome or chromosomes. If this was the case, we concluded that the cases were confirmed and indicated the parental chromosome(s) that were absent. For triploid and trisomy cases, the SNPs with opposing homozygotes in the parents were investigated. For those SNP, we checked whether the BAF of the embryo was above or below 0.5, with below 0.5 assuming 2 copies of A and one copy of B (AAB), and above 0.5 assuming one copy of A and 2 copies of B (ABB). For each SNP for which the parents had opposing homozygotes, this indicated from which parent the extra copy was inherited at that position. If that was the same across the whole chromosome(s), we concluded that the cases were confirmed and indicated the parents of which the chromosome(s) were present with an extra copy.

The quality of embryo genotypes is in general lower than genotypes of live-born individuals. The embryo genotypes showed lower call rates and larger standard deviations of BAF and LRR than samples from live-born individuals (24 hair samples from cows genotyped in same batch; results not shown). This noise is likely due to the pre-amplification of the limited amount of DNA from the few cells taken from the embryos. For all 558 embryos, the call rate ranged between 0.256 and 0.987, with an average of 0.844 (±0.15).

Embryos with a call rate below 0.8 were screened for unbalanced aberrations but excluded from further analysis. They were screened because some aneuploidy events (e.g., mosaic, triploid, or nullisomy) can cause low call rates. Of the 118 embryos with call rate below 0.8, 67 failed due to technical issues (only AA, only BB, or only AB calls), and the remaining 51 showed over-dispersed heterozygotes (discussed below). No unbalanced aberrant cases were identified in embryos with a call rate below 0.8, although issues could have been present but remained undetected due to poor genotype quality.

The average call rate of the 440 embryos with call rate above 0.8 was 0.910 (±0.05). Among these 440 embryos, 10 failed quality control. They showed only BB calls, which was a technical issue. In total, 246 samples were classified as normal, 22 samples as abnormal (Table 1), and the remaining 162 samples showed over-dispersed heterozygotes. These over-dispersed samples have large clusters of heterozygotes on many or all chromosomes, but no clear clusters within the heterozygote cluster as polyploidy would have. Hence, these over-dispersed samples were difficult to classify, as they could be technical errors (e.g., due to amplification of DNA) but could also be cases of polyploidy (e.g., due to polyspermy) or cases of mosaicism. Polyspermy is known to occur in IVF embryo production (reviewed in Coy and Avilés, 2010), and mosaicism is also rather commonly observed in embryos (Fragouli et al., 2019). We found it very hard to differentiate potential polyspermy or mosaicism from technical issues, as the genotype intensity signals of the embryos were rather noisy in general. In our cases all, or at least many, chromosomes were affected, and therefore the cause was less likely to be mosaicism but, given the large number of samples, most likely a technical issue.

**Table 1:** Detected cases with unbalanced chromosomal aberrations; parental origin and pregnancy status if transplanted

Sample	Issue	Affected chromosome	Sex	Parental origin^1^	Call rate	Status^2^
A	Haploid	All	Y	Maternal genome absent	0.94	NP
B	Haploid, nullisomy BTA10	All	X	Maternal genome absent	0.84	NP
C	Monosomy	6	XX	Maternal chromosome absent	0.87	BA
D	Monosomy	16	XY	Paternal chromosome absent	0.91	NT
E	Monosomy	6	XX	Paternal chromosome absent	0.92	NT
F	Monosomy	15	XY	Maternal chromosome absent	0.92	NT
G	Monosomy	25	XY	Maternal chromosome absent	0.93	NT
H	Monosomy	1	XY	Maternal chromosome absent	0.94	NT
I	Monosomy	4	XX	Maternal chromosome absent	0.94	NT
J	Monosomy	1	XX	Maternal chromosome absent	0.94	NT
K	Monosomy	21	XY	Paternal chromosome absent	0.95	NT
L	Monosomy	12	XX	NA	0.95	NP
M	Monosomy	15	XY	Maternal chromosome absent	0.95	NT
N	Monosomy	14	XY	NA	0.96	NP
O	Monosomy	14	XY	Maternal chromosome absent	0.97	NT
P	Monosomy	9	XY	Maternal chromosome absent	0.97	NT
Q	Triploid	All	XXX	Extra maternal copy	0.85	NT
R	Triploid	All	XXY	Extra maternal copy	0.85	NT
S	Triploid	All	XXY	Extra maternal copy	0.85	NT
T	Triploid	All	XXY	Extra maternal copy	0.86	NT
U	Trisomy	24	XY	NA	0.96	NT
V	Trisomy	14, 19	XY	Extra maternal copies	0.96	NT

1 Parental origin designated NA for cases without genotypes of (both) parents, for which parental origin could not be determined.

2 Status = pregnancy status, with NT indicating embryo was not transferred to recipient; NP means not pregnant; BA means that pregnancy was successful and the resulting calf was born alive.

We found 2 haploid cases, 14 monosomy cases, 4 triploid cases, and 2 trisomy cases (Table 1); examples are given in Figure 1. Although the number of unbalanced cases is limited, BTA 1, 6, 14, 15 were impaired multiple times (Table 1). Silvestri et al. (2021) showed that BTA 14 is highly susceptible to chromosomal issues, and also BTA 1, 6, and 15 were more prone to errors among others. The overall call rate of the ploidy issue cases ranged from 0.84 to 0.86, and of monosomy and trisomy cases from 0.87 to 0.97, indicating that overall call rates are not necessarily impaired (Table 1).

**Figure 1:**
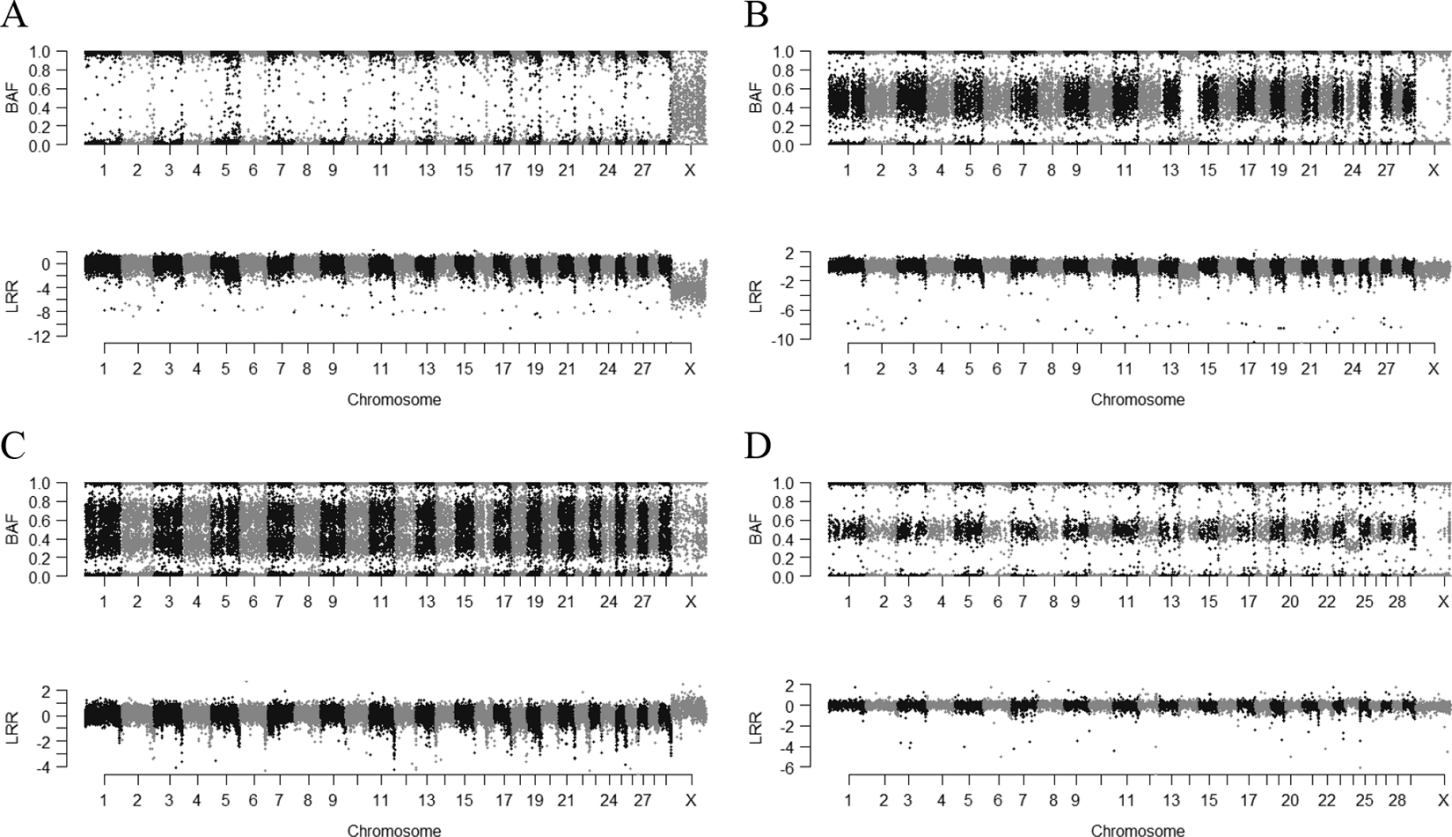
B-allele frequency (BAF) and log R ratio (LRR) plots of cases with unbalanced chromosomal aberrations. (A) Haploid case (A in Table 1), where the heterozygote cluster around BAF = 0.5 is lacking for all autosomes, and the non-pseudo autosomal region of the X chromosome is completely missing (hence the LRR around −5). (B) Monosomy case (O in Table 1), where the heterozygote cluster around BAF = 0.5 is lacking for BTA14, where we also see a drop in LRR. (C) Triploid case (Q in Table 1), where 2 heterozygote clusters are present for all autosomes, as well as for the X chromosomes, one around 0.33 for AAB genotypes and one around 0.66 for ABB genotypes. (D) Trisomy case (U in Table 1), where 2 heterozygote clusters are present for BTA24 around 0.33 and 0.66, and the LRR is higher for BTA24 compared with the other chromosomes.

Strikingly, both haploid cases lacked the maternal genome (Table 1); unfortunately the numbers are too small to suggest causal mechanisms. Tutt et al. (2021) found a haploid case lacking the paternal genome, suggesting parthenogenesis. The triploid cases were rather clear and were likely noted while determining the sex based on SNP genotypes and hence not implanted. Of the 4 triploids, 3 were full sib embryos from the same oocyte harvesting. All 4 were shown to contain an extra maternal copy (Table 1). For 3 cases (L, N, and U in Table 1) parental origin could not be determined because genotypes of both parents were lacking. In 16 out of 19 cases it was the maternal chromosome(s) that was lost or gained; for only 3 monosomies the paternal chromosome was lost (Table 1).

The results indicate a 5% incidence rate of unbalanced chromosomal aberrations, taking 430 successfully genotyped stage 8 embryos into account, and even 8% when considering only the 268 samples properly classified (discarding the over-dispersed heterozygotes samples). Either way, the incidence rate of unbalanced chromosomal aberrations is somewhat higher than the 2% reported by Silvestri et al. (2021) based on only 103 stage 8 to 9 embryos. Caution should be taken in comparing these incidences to human embryo studies, as in cattle the oocytes are matured in vitro, whereas that is not common practice in humans. In vitro maturation leads to a higher incidence, compared with in vivo-derived embryos (Tšuiko et al., 2017; Tutt et al., 2021).

The embryos are genotyped for genomic selection, and only the embryos with the best genomic breeding values are implanted. Only 5 of the affected cases were implanted in recipients. Of these, 4 did not lead to pregnancies (Table 1). Remarkably 1 of them, with monosomy on BTA6 (Figure 2A), was born alive (Table 1). This individual was re-genotyped as a live-born individual and proved to be diploid for all chromosomes, including BTA6 (Figure 2B). The genotypes of the live-born individual and its embryo showed good concordance in genotype calls on all chromosomes, except BTA6 (Figure 2C); the correlation between genotypes was 0.79 for BTA6 and 0.98 for the remaining chromosomes. This indicates that there was no sample mix-up and suggests that the embryo developed further into a healthy diploid individual. Silvestri et al. (2021) also noted that some embryos identified as aneuploid led to live-born calves, and the same has been observed in humans (Rubino et al., 2016). For mosaic embryos with euploid and aneuploid cells, it seems that the aneuploid cells are progressively depleted from the embryo as it develops, and such embryos have full developmental potential; however, embryos with 100% aneuploid cells were unviable (Bolton et al., 2016; Yang et al., 2021). Hence, a possible explanation may be mosaicism, where aneuploid cells are segregated in the trophectoderm of the embryo, while the inner cell mass contained euploid cells.

**Figure 2:**
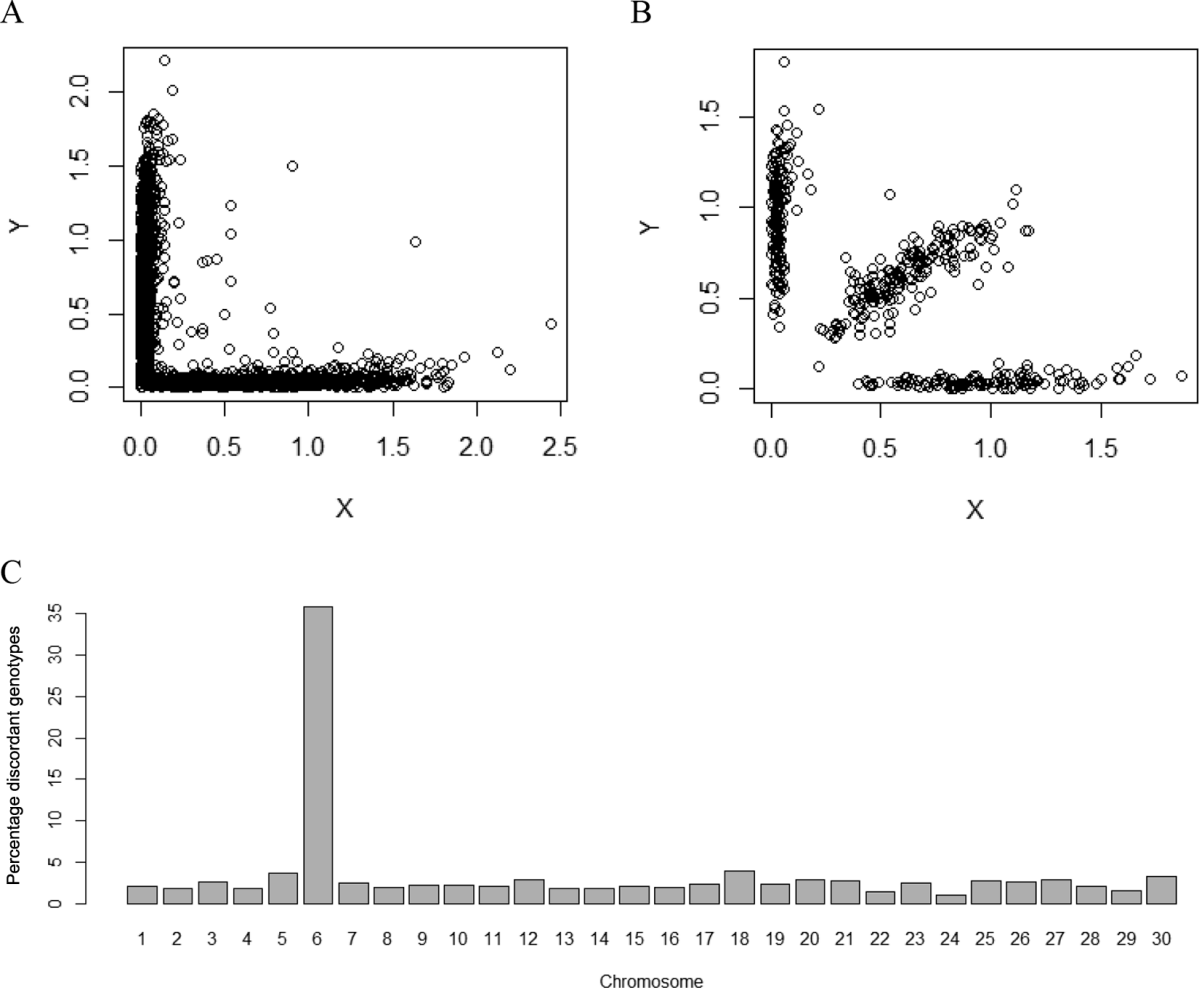
Differences in genotypes between monosomic embryo and its live-born calf. (A) X and Y intensity plot of BTA6 of embryo with monosomy BTA6 (C in Table 1) showing only 2 genotype clusters (genotypes A and B). (B) X and Y intensity plot of BTA6 of the live-born calf. The calf has 3 genotype clusters (genotypes AA, AB, BB) indicating a diploid BTA6. (C) Bar graph showing the percentage of discordant genotypes between the monosomy embryo and its live-born calf per chromosome. Although some mismatches across the genome occur due to incorrectly genotyped SNPs, it is much larger at BTA6.

Given that the embryos in dairy cattle breeding programs are genotyped for other purposes, it is relatively easy and inexpensive to screen them for unbalanced chromosomal aberrations. In addition, only a limited number are selected for transfer (i.e., the ones with the best breeding values); hence, there is room to select only the embryos that appear diploid on all chromosomes. Therefore, preimplantation genetic testing is practically feasible. This study was an attempt to provide insight in the incidence of unbalanced chromosomal aberrations. Only a limited number of cases detected in current study were transferred into recipients (5/22); hence, we cannot predict increases in pregnancy success rate if detected embryos are excluded from transfer—especially because one monosomy case gave rise to a diploid live-born calf, whereas, on the other hand, both transferred haploid cases did not lead to pregnancies. In time, larger data sets can be accumulated by screening, which will provide better insight on the incidence and characteristics of unbalanced chromosomal aberrations. At the same time, specific experiments may be needed to gain knowledge on the representativeness of a small number of blastocyst cell for the inner cell mass or even the produced offspring. Here cattle can be (and currently are being) used as a model species to upscale human studies, which are often limited due to ethical reasons. This will enhance our knowledge and lead to sufficient data for better-informed decisions for embryo transfer programs, in both cattle and humans.

To conclude, we found a 5% incidence rate of unbalanced chromosomal aberrations in a retrospective study among 430 genotyped cattle embryos. Monosomy was most frequently observed. Given that embryo genotypes are readily available, monitoring incidence can be applied. Moreover, selection for euploid embryos without ploidy issues may improve pregnancy rates for in vitro embryo transfer.
